# Impact of a high-fat meal on assessment of clopidogrel-induced platelet inhibition in healthy subjects

**DOI:** 10.1186/s12959-014-0033-x

**Published:** 2015-01-23

**Authors:** Paul P Dobesh, Jamela F Urban, Scott W Shurmur, Julie H Oestreich

**Affiliations:** Department of Pharmacy Practice, University of Nebraska Medical Center College of Pharmacy, Omaha, Nebraska 68198-6045 USA; Pharmacy Department, Denver Health Medical Center, Denver, Colorado USA; Division of Cardiology, Texas Tech University Health Science Center, Lubbock, Texas USA; University of Kentucky College of Pharmacy, Lexington, Kentucky USA

**Keywords:** Clopidogrel, Blood platelets, Platelet function tests, P2Y12 purinoceptor antagonist, Diet, High-fat

## Abstract

**Background:**

Ideal conditions for platelet reactivity testing are critical for optimal selection of a P2Y12 inhibitor. Data are inconsistent regarding the impact of high-fat meals on test assessment.

**Methods:**

Participants included 12 healthy subjects not taking antiplatelet drugs after a 12-hour fast. After baseline assessment, subjects were given a 600 mg dose of clopidogrel. Four hours later, maximum platelet inhibition was tested in the fasting state by light transmission aggregometry (LTA), VerifyNow P2Y_12_, vasodilator-stimulated phosphoprotein (VASP), and whole blood aggregometry (WBA). Subjects were then provided a high-fat meal, and platelet function was evaluated two hours later. Change in measured platelet aggregation by LTA was the primary endpoint of the study. The Wilcoxon Rank Sum test was used to compare the change in platelet reactivity between fasting and non-fasting conditions. The Spearman rho (ρ) correlation coefficient was used to evaluate the association between fasting platelet reactivity and the change following a high-fat meal.

**Results:**

No significant change occurred in maximal light transmission, as assessed by LTA with 5 μM ADP (p = 0.15) and with 20 μM ADP (p = 0.07). There was a significant change in the area under the curve with 5 μM ADP (p = 0.03) but not with 20 μM ADP (p = 0.18). Although there was no significant change with the VerifyNow P2Y12 assay (p = 0.16), the change was correlated with the initial fasting value (Spearman’s rho p = 0.008). The VASP assay and WBA varied minimally.

**Conclusion:**

The high-fat meal did not significantly alter platelet function assessment of commonly used platelet function tests. Greater intra-subject variability existed for the optically-dependent compared with non-optically dependent tests.

**Trial registration:**

NCT01307657.

## Background

The antiplatelet agent clopidogrel is commonly prescribed in the United States and around the world for the treatment of cardiovascular disease. A number of large, randomized clinical trials have demonstrated the clinical efficacy of clopidogrel and the importance of its ability to irreversibly antagonize P2Y_12_ receptors on platelets [1-4]. Through inhibition of the P2Y_12_ receptor, clopidogrel prevents platelet activation and aggregation, leading to a reduction in arterial thrombotic events, such as myocardial infarction and stroke in patients with cardiovascular disease.

Although clopidogrel has demonstrated clinical efficacy, multiple factors contribute to high patient variability in response to the drug, such as acute coronary syndrome, body mass index, diabetes mellitus, adherence, drug interactions, and genetics [5]. Importantly, patients with high on-treatment platelet reactivity while taking clopidogrel demonstrate a significant increase in major adverse cardiac events compared to patients with normal or decreased platelet reactivity while taking clopidogrel [6]. Even with the known variability in patient response to the drug, clopidogrel is prescribed as a standard dose for all patients. Although controversial, some evidence suggests that it may be advantageous and cost-effective in some situations to personalize antiplatelet therapy based on pharmacodynamic platelet assessment [7]. Measuring platelet response may also help determine if newer options for antiplatelet therapy are more appropriate.

While other P2Y_12_ receptor antagonists (i.e. prasugrel and ticagrelor) provide more potent inhibition of platelet aggregation and reduced ischemic outcomes compared to clopidogrel, these medications are also considerably more expensive than generic clopidogrel and result in increased rates of major bleeding [8,9]. While the ideal management strategy for patients with high on-treatment platelet reactivity is under debate, knowledge of a patient’s response to clopidogrel has the potential to direct treatment decisions and help improve clinical outcomes.

Currently, platelet function tests are not standardized among institutions [10]. Furthermore, while controversy exists regarding the ideal test to use for monitoring clopidogrel therapy, even less is known about optimal testing conditions [11]. One vital testing variable that has not been well-studied for all assays is whether an accurate assessment of platelet reactivity assessment depends on fasting status. Data on the impact of food on platelet function assessment are quite inconsistent in terms of methodology and results [12].

Previous studies reveal conflicting results concerning the effect of fasting and non-fasting conditions as well as whether or not a high-fat meal has an effect on the assessment of platelet aggregation [13-21]. Multiple factors could explain a potential effect of a high-fat meal on platelet aggregation assessment. For example, one physiologic mechanism involves platelet interactions with triglycerides and chylomicron remnants that are present in the bloodstream after a high-fat meal. It has been suggested that platelet uptake of triglycerides induces release of platelet factor 4 and promotes platelet aggregation. Additionally, it has been implied that the chylomicron layer on the platelet itself may interfere with the interaction between platelets and collagen [15,22,23]. Another possibility relates to the increased cloudiness of a lipemic sample that a high-fat meal causes. This cloudiness of the sample possibly hinders the ability of optically dependent assays to properly measure the extent of platelet aggregation. Regardless, most previous studies that evaluate the effect of a high-fat meal on platelet aggregation have used light transmittance aggregometry (LTA), but other platelet assays increasingly used in clinical practice (e.g. VerifyNow) are underrepresented in current studies [11,12].

The purpose of the study herein was to determine the influence of a high-fat meal on the assessment of platelet function by comparing several different assays available. With the increasing study and utilization of platelet function testing in clinical practice, determining the appropriate testing conditions (fasting vs. non-fasting) is critical. Furthermore, the ability to appropriately assess platelet response to clopidogrel therapy will assist in determining the optimal treatment strategy for reducing ischemic outcomes and minimizing bleeding in patients with cardiovascular disease.

## Methods

The study was approved by the Ethics Committee of the University of Nebraska Medical Center (IRB 568-10), and all subjects demonstrated willingness to participate through understanding and signing a consent form in accordance with the principles of the Helsinki Declaration. Twelve healthy adults not taking clopidogrel were recruited for this prospective, single-center study. Likewise, all volunteers denied taking any medications that affect platelet function for at least seven days prior to participation in the study. Volunteers were excluded if they had cardiovascular disease or modifiable risk factors for cardiovascular disease (i.e., hypertension, dyslipidemia, diabetes mellitus, or smoking), a history of renal or hepatic disease, illnesses requiring hospitalization or surgery within the last three months, a history of anemia or thrombocytopenia, currently taking proton pump inhibitors, and/or a history of excessive bleeding or thrombosis. Women who were pregnant were also excluded.

**Figure 1:**
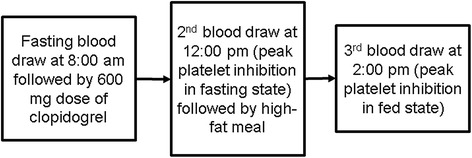
**Study design.**

Information gathered in this study was used to assess if the presence of a high-fat meal alters the effectiveness of several different types platelet function tests to measure platelet inhibition by clopidogrel (Figure 1). Following a 12-hour fast, subjects were presented to the Clinical Research Center of the University of Nebraska Medical Center at 8:00 a.m. A baseline, fasting blood sample was drawn, and subjects were then administered a 600 mg dose of clopidogrel taken with water. This loading dose of clopidogrel is commonly used in clinical practice and achieves maximal inhibition of platelet reactivity in two to four hours, which is sustained for at least six hours [24]. At 12:00 p.m. (four hours after clopidogrel dosing), another blood sample was drawn to evaluate the extent of maximum platelet inhibition in the fasting state. Following the second blood draw, subjects were administered a standardized high-fat meal of a fast food hamburger and french fries that consisted of 54 grams of fat (79% daily value), 13.5 grams of saturated fat (65% daily value), 108 grams of carbohydrate (38% daily value), 31 grams of protein, and a total caloric content of 1,040. The third blood sample was drawn at 2:00 p.m., two hours following completion of the high-fat meal, to evaluate the impact of the high-fat meal on the platelet reactivity assessment.

Complete blood counts (CBC) were obtained at the baseline blood draw, and lipid panels were evaluated at fasting (12:00 pm) and at 2:00 pm following the high-fat meal. Whole blood was collected from a venous source into anti-coagulated tubes (3.2% sodium citrate) at the following three blood collection time points: fasting baseline (non-medicated) (8:00 am), peak platelet inhibition in the fasting state (12:00 pm), and peak platelet inhibition after the high-fat meal (2:00 pm). Platelet function was assessed at each time point using four different types of platelet inhibition assays, which included two optically-dependent assays—LTA and the VerifyNow® P2Y12 assay (Accumetrics, San Diego, CA)—and two non-optically dependent assays—flow cytometry (VASP [vasodilator-stimulated phosphoprotein ] P2Y12 assay, GP IIb/IIIa receptor activation, and CD62P expression) and whole blood impedance aggregometry.

### Light transmittance aggregometry

Samples of platelet-rich plasma (PRP)—obtained by centrifugation of whole blood at 100 g for 15 minutes—were incubated for three minutes in glass cuvettes and then activated by 5 μM and 20 μM adenosine diphosphate (ADP) in a two-well Model 700 aggregometer (Chrono-Log) under stirring conditions. Platelet-poor plasma was collected after centrifugation at 2400 g for 20 minutes and placed in the reference well to establish the baseline transmittance for each subject. Aggregometry samples were monitored for six minutes; the area under the curve (AUC) and amplitude were calculated using AGGRO/LINK 8 software (version 1.2.3, Chrono-Log).

### VerifyNow® P2Y12 Assay

Whole blood collected in 2 mL citrated Greiner vacuette tubes (Bio-One) was gently inverted and inserted onto P2Y12 cartridges for analysis using the VerifyNow® device. Prior to testing, an electronic quality control was performed daily. Data were reported as P2Y12 Reaction Units (PRU).

### VASP phosphorylation assay

Flow cytometry was used to monitor phosphorylation of VASP, an intracellular platelet protein specific for the P2Y_12_ pathway, per the recommended guidelines for the platelet VASP/P2Y12 kit (Biocytex, Marseille, France). Whole blood was incubated with prostaglandin E1 either alone or with ADP. The samples were fixed and then incubated with a permeabilization agent and antibodies specific to phosphorylated VASP. A separate sample was used to test a negative isotypic control antibody. Next, fluorochrome-labeled antibodies CD61-PE and polyclonal IgG- PAC1-fluorescein (FITC) were added to each sample to label platelets and anti-VASP antibodies, respectively. Platelets were identified by scatter (forward and side) and PE fluorescence. Data were captured for at least 5,000 platelet events using the FACSCalibur system. The geometric mean fluorescence intensity (MFI) was determined with CellQuest Pro software (Version 5.2.1, Becton, Dickinson and Company), and the platelet reactivity index (PRI) was calculated using the following formula: PRI = [(MFI_PGE1_ – MFI_(PGE1+ADP)_) / MFI_PGE1_] × 100.

### Flow cytometry of PAC1 and CD62P

To further assess platelet activation by using flow cytometry, the activated GP IIb/IIIa receptor was specifically targeted with PAC1, and CD62P (P-selectin), which is selectively expressed on the platelet surface after platelet alpha granule release, as previously described [25,26]. Whole blood was diluted 1:7 (v/v) in a modified Tyrode’s buffer (137 mM NaCl, 2.8 mM KCL, 1 mM MgCl_2_, 12 mM NaHCO_3_, 0.4 mM Na_2_HPO_4_, 10 mM HEPES, 0.35% bovine serum albumin, and 5.5 mM glucose; filtered, pH 7.4). Samples were incubated at 37°C with platelet agonist or buffer as a resting control. Next, the following fluorochrome-labeled antibodies were added at room temperature protected from light: FITC, CD62P-allophycocyanin (APC), and CD42b-phycoerythrin (PE; all from Becton, Dickinson and Company). CD42b is a surface marker for platelets. IgM-FITC and IgG-APC were used as antibody controls. Next, samples were fixed with 1% paraformaldehyde, diluted with buffer, and processed at the Cell Analysis Facility at the University of Nebraska Medical Center. The samples were analyzed with CellQuest Pro software (Version 5.2.1). Platelets were identified by scatter (forward and side) and PE fluorescence, and data were captured for 10,000 platelet events. Platelet activation was determined by geometric MFI.

### Whole blood impedance aggregometry

Samples of whole blood (500 μL) were diluted 1:1 (v/v) with normal saline and evaluated by impedance aggregometry in a two-well Model 700 aggregometer (Chrono-Log). Samples were pre-warmed for three minutes in the incubation wells. Under stirring conditions (1200 rpm), aggregometry samples were processed for six minutes following stimulation by ADP 5 and 20 μM.

### Statistical analysis

Change in measured platelet aggregation by LTA was the primary endpoint of the study. Based on previous data for patients treated with clopidogrel at enrollment and retested 60 days later (SD = 14; r = 0.68) [27], the two-sided paired t-test was used to estimate the needed sample size. Thus, n = 12 people was an appropriate sample size to identify a 10-unit change in LTA at 80% power with alpha set at 0.05. The Wilcoxon Rank Sum test was used to compare the change in platelet reactivity (between fasting and non-fasting conditions) to zero after treatment with clopidogrel. The Spearman rho (ρ) correlation coefficient was used to evaluate the association between fasting platelet reactivity and the change following a high-fat meal. Also, the statistical package SPSS 19 (IBM) software was used, and the level of significance was set at 0.05.

## Results

The mean age of the seven males and five females recruited to participate in the study was 35 ± 9 years. All portions of the CBC profiles were within normal limits. At the time of the fasting blood sample after treatment with clopidogrel (12:00 pm), subjects had lipid profiles within the normal limits (Table 1). At two hours following intake of the standardized high-fat meal (2:00 pm), there was a significant (67%) increase in triglycerides from 79 mg/dL to 132 mg/dL (p = 0.002), which indicates that a lipemic state had been achieved in these two hours. The other lipid markers were similar between the fasting and non-fasting states after treatment with clopidogrel (Table 1).

**Table 1:** **Lipid panel for the on-clopidogrel fasting and non-fasting (post-high fat meal) states**

**Lipid panel**	**Fasting**	**Post-meal**
Total Cholesterol (mg/dL)	181 ± 37	172 ± 41
Triglycerides (mg/dL)	79 ± 52	132 ± 100*
LDL-C (mg/dL)	113 ± 23	95 ± 25
HDL-C (mg/dL)	53 ± 19	50 ± 18
VLDL-C (mg/dL)	16 ± 10	21 ± 8
Cholesterol/HDL-C	3.7 ± 1.1	3.5 ± 0.8

*p = 0.002.

*LDL*-*C* = low density lipoprotein cholesterol; *HDL*-*C* = high density lipoprotein cholesterol; *VLDL*-*C* = very low density lipoprotein cholesterol.

**Figure 2:**
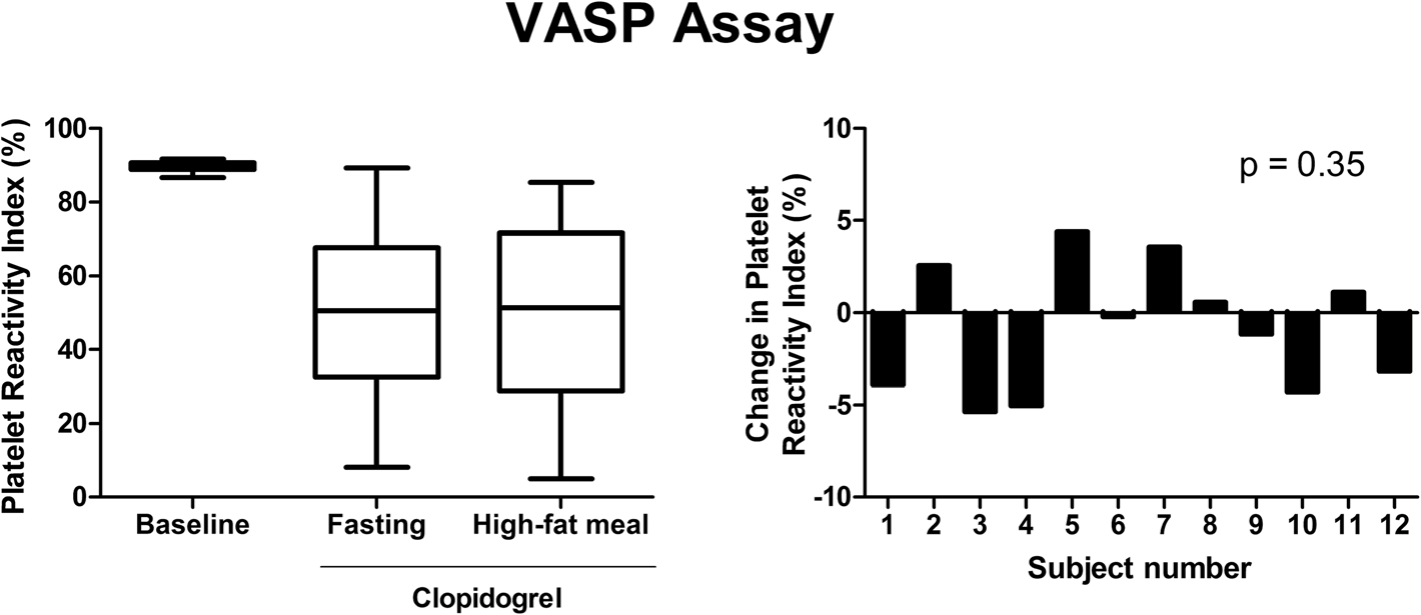
**Effect of a high-fat meal on the VASP P2Y12 assay.** The change in platelet reactivity index from the on-treatment fasting to non-fasting state was not significantly different from zero (p = 0.35). n = 12.

Clopidogrel-induced platelet inhibition, as measured by flow cytometry, was consistent among the fasting and non-fasting conditions following the dose of clopidogrel (Figure 2). For the VASP P2Y12 assay, the median PRI for the fasting state following the clopidogrel dose was 51% (range = 8-89%), compared to 51% (range = 5-85%) for the non-fasting state. The change in PRI between the fasting and non-fasting conditions following the clopidogrel dose was not significant from zero (p = 0.35) and indicated low variability. The median change in PRI after the high-fat meal was 3% (range = 0-5%) after adjustment for the absolute value of change. Similarly, the results for flow cytometric analysis of CD62P (p = 0.70 and 0.64 for ADP 5 μM and 20 μM, respectively) and PAC1 (p = 0.27 and 0.24 for ADP 5 μM and 20 μM, respectively) support no significant difference in platelet activation after treatment with clopidogrel and following the high-fat meal (data not shown).

**Figure 3:**
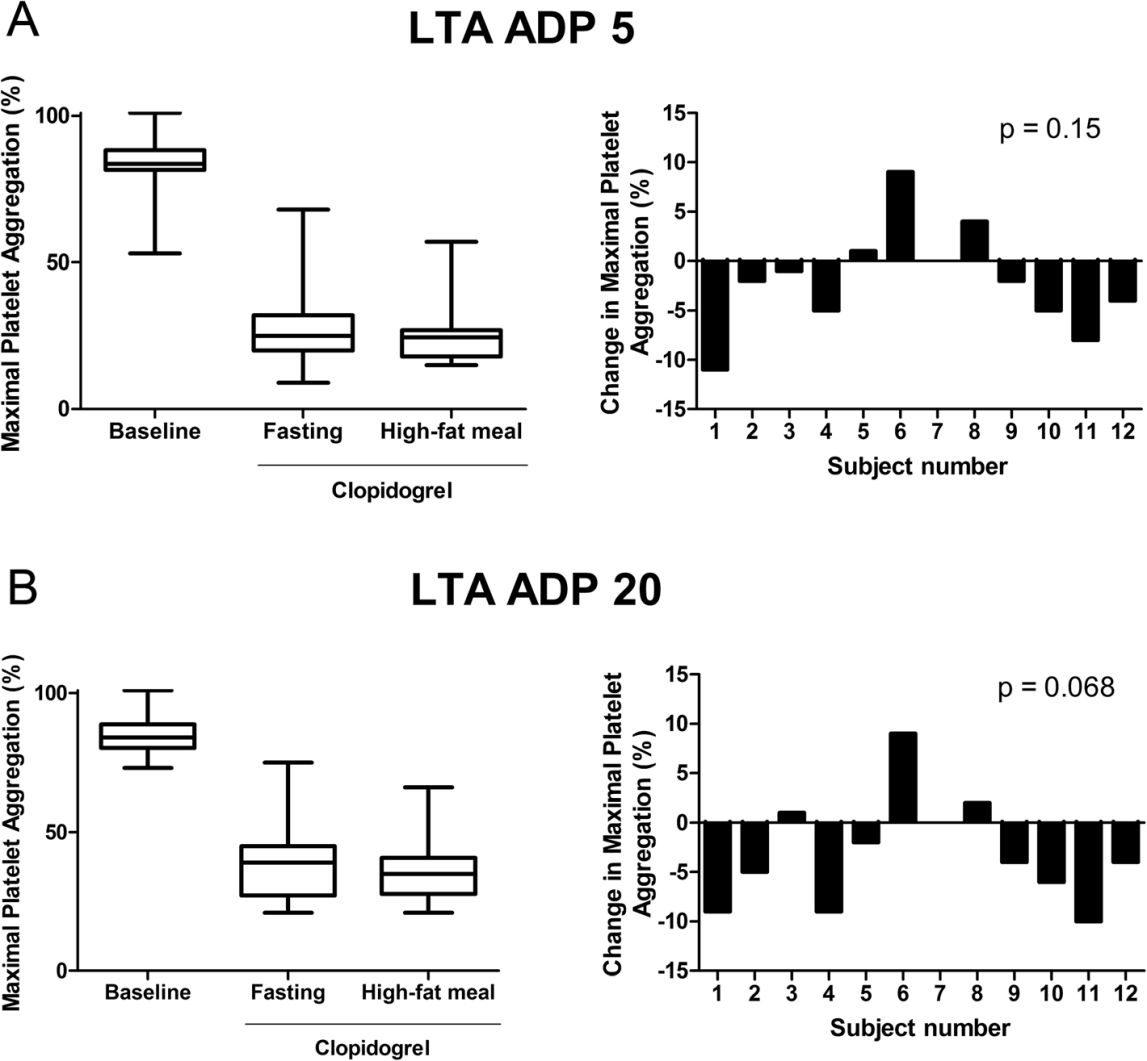
**Impact of a high-fat meal on ADP-stimulated light transmittance aggregometry.** The change in maximal platelet aggregation on-clopidogrel from the fasting state compared to following the high-fat meal was not significantly different from zero for both concentrations of ADP, 5 μM (p = 0.15) and 20 μM (p = 0.068). n = 12.

The optically-based LTA assay demonstrated no significant change in platelet aggregation between the fasting and non-fasting states after the dose of clopidogrel for both 5 μM (p = 0.15) and 20 μM (p = 0.068) ADP (Figure 3). A small, but significant change was identified in the AUC with 5 μM ADP (p = 0.034) as the median AUC decreased from 26 (range = 4-444) to 18 (range = 6-380) from the fasting to non-fasting time points after treatment with clopidogrel (data not shown). However, no significant change was apparent for ADP 20 μM (p = 0.18).

**Figure 4:**
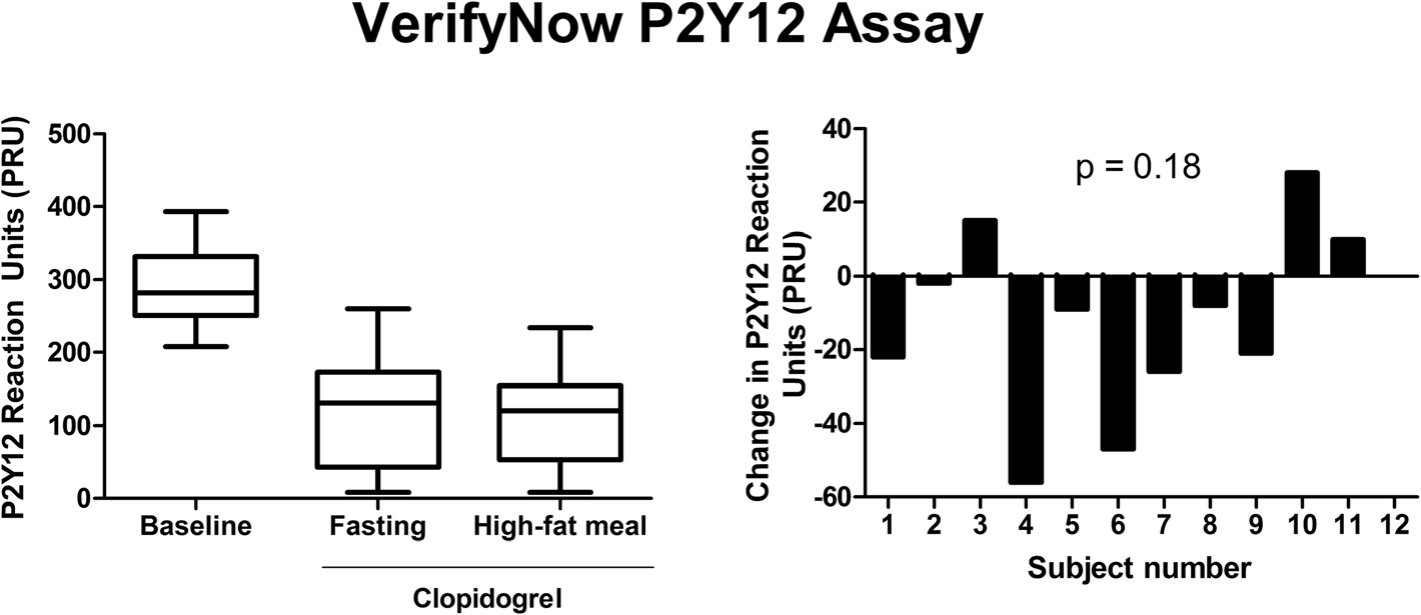
**Impact of a high-fat meal on the VerifyNow P2Y12 assay.** The change in P2Y12 Reaction Units (PRU) was not significantly different from zero (p = 0.18). n = 12.

**Figure 5:**
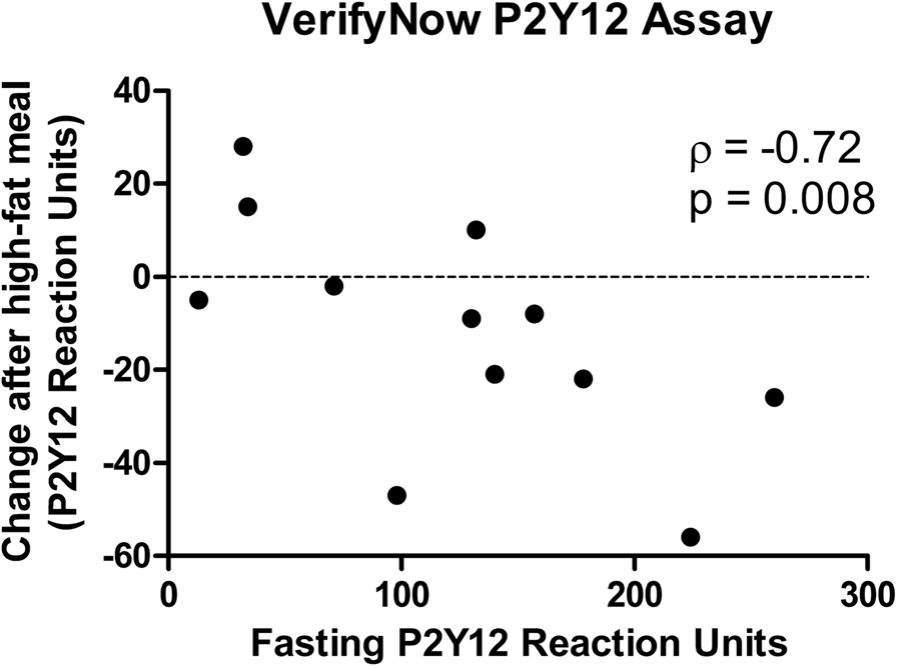
**Correlation between fasting on-clopidogrel platelet reactivity and the change in on-treatment platelet reactivity after a high-fat meal for the VerifyNow P2Y12 assay.** Spearman correlation coefficients (ρ) were generated for each assay to assess the correlation between: 1) fasting on-clopidogrel platelet reactivity and 2) the change in platelet reactivity on-clopidogrel from the fasting to the non-fasting state. The significant finding for the VerifyNow P2Y12 assay (p = 0.008) suggests that subjects with high platelet reactivity on clopidogrel may be selectively impacted by the high-fat meal, while subjects with a normal clopidogrel response may have more consistent results regardless whether or not a high-fat meal is taken.

Congruent with the other assays, there was no significant change in platelet inhibition using the VerifyNow P2Y12 assay to compare the fasting to non-fasting state (p = 0.18) (Figure 4). Despite this finding, notable variability displayed as the fasting to non-fasting change in PRU values ranged from -56 PRU to 28 PRU. Interestingly, subjects with higher platelet reactivity in the fasting state after treatment with clopidogrel tended to have decreased PRU values after intake of the high-fat meal, while those with lower platelet reactivity primarily demonstrated limited changes or increased PRU values in the non-fasting state (ρ = -0.72; p = 0.008) (Figure 5). A similar trend was demonstrated with LTA and 5 μM ADP (ρ = -0.53; p = 0.08) and with 20 μM ADP (ρ = -0.54; p = 0.069), but neither reached statistical significance.

Complete platelet inhibition was demonstrated in eight of the 12 subjects with whole blood aggregation. Therefore, changes in platelet aggregation due to the impact of a high-fat meal were not easily assessed with this method (data not shown).

## Discussion

In this study, intake of a high-fat meal did not have a statistically significant impact on the results of four commonly used platelet function tests. The only significant change was demonstrated in the aggregation AUC with LTA using 5 μM ADP when comparing clopidogrel platelet inhibition during fasting and after a high-fat meal. Although there was no change in platelet reactivity with the VerifyNow P2Y12 assay, a significant correlation existed between fasting platelet reactivity with clopidogrel and the change in platelet reactivity from fasting to after the high-fat meal. This result suggests that subjects may demonstrate a greater change to the high-fat meal with high platelet reactivity while taking clopidogrel compared to those subjects with normal clopidogrel response.

Previous research on the impact of a high-fat meal and platelet function assessment has produced conflicting results. Some studies demonstrate an increase in baseline, non-medicated platelet aggregation in patients after chronic or long-term ingestion of a high-fat diet. Still, the differences in platelet aggregation in these studies are fairly minimal and are confounded by other variables such as the type of fat ingested, patient comorbidities, and methods for assessing platelet function [14,28,29]. For example, studies by Nordøy and associates demonstrate an increase in platelet aggregation after acute ingestion of a high-fat meal. However, in these studies the fat content of the meal reached 100 to 175 grams, provided by ingesting whipping cream [13,15]. This represents a two- to three-fold increase in fat content compared to the more typical high-fat meal provided in this current study. Conversely, Fuhrman and associates were able to demonstrate an absolute increase in platelet aggregation of 3% with acute ingestion of roughly 50 grams of fat [18]. Therefore, meaningful increases in platelet aggregation are not demonstrated without extreme fat intake.

Similar to the present study, there have also been a number of studies that demonstrate no notable change in platelet aggregation after acute ingestion of a high-fat meal [16,17,19-21]. Unlike the present study, these studies did not evaluate multiple platelet aggregation assays or have subjects receiving an antiplatelet agent. Contrary to the studies by Nordøy and associates, the consistent lack of an impact on platelet aggregation was demonstrated regardless of the amount of fat in the high-fat meal, even at levels greater than 100 grams per meal [16,19]. There are a number of possible explanations of why some studies report an increase in platelet aggregation and others do not. One possible explanation is that there is actually a protective layer of chylomicrons that form on platelets after a high-fat meal that prevent platelet activation [23,30]. Because most studies demonstrating an increase in platelet aggregation after a high-fat meal evaluated washed platelets, it is possible that this protective layer was removed in this process. There have also been studies that show that as opposed to PRP, washed platelets increase collagen and ADP aggregation after fat intake. Another limitation to the research demonstrating an increase in platelet aggregation after acute ingestion of a high-fat meal is the use of heparin-neutralizing activity as an indicator for plasma platelet factor 4 levels and thereby platelet activity. Considering that there are numerous platelet-independent factors that have heparin neutralizing activity, the use of platelet factor 4 as an endpoint is questionable [16]. The present study avoids this limitation because PRP was used for LTA and not washed platelets, as well as commonly used measures for platelet aggregation.

Although these previous studies suggest a potential physiologic interaction of platelets with triglycerides, the results of the present study do not support this hypothesis. Based on the four platelet function tests evaluated, a high-fat meal was not found to significantly impact platelet aggregation assessment. Due to the limitations discussed in studies demonstrating an increase in platelet aggregation after acute ingestion of a high-fat meal, and the larger body of evidence suggesting a lack of influence of a high-fat meal on platelet aggregation, any variability demonstrated with the optically-dependent assessment of platelet function are unlikely due to a physiologic interaction of platelet with triglycerides. If this type of interaction did occur, it would be expected to be detected as changes in platelet aggregation in the VASP assay in the present study. Instead, the exceptional consistency of the VASP results before and after the high-fat meal disputes this possibility. Although there was not an impact of a high-fat meal on most platelet function tests used in this study, the optically dependent tests demonstrated more intra-subject variability compared to the non-optically dependent test.

The impact of a high-fat meal with clopidogrel has been evaluated in other studies with different designs compared to the present study [31,32]. For example, one of these studies only evaluated the impact of the high-fat meal on the bioavailability and pharmacokinetics of clopidogrel [31]. While this study did not find a significant change in the C_max_, T_max_, or AUC for clopidogrel, this study also did not evaluate the influence of the high-fat meal on the pharmacodynamics of clopidogrel. The study by Hurbin and associates found a non-significant 4.7% difference in maximal platelet aggregation before and after a high-fat meal as assessed by LTA with 5 μM ADP. Unlike the present study, the evaluations of the non-fasting and fasting states were not compared on the same day two hours apart, but instead they were compared two weeks apart. Their study also evaluated a loading dose of clopidogrel 300 mg compared to the commonly used dose of 600 mg in the present study. The present study significantly adds to these data because the more commonly used VerifyNow® assay and VASP assay were evaluated, as well as the timely impact of a high-fat meal to assessment of platelet aggregation.

Based on a thorough review of the literature, the present study is the first to evaluate the impact of a high-fat meal on clopidogrel-induced platelet aggregation with both optically dependent and non-optically dependent assays. Subjects were evaluated at baseline and after receiving a 600 mg dose of clopidogrel, which provides more clinically useful relevant information compared to evaluating volunteers not taking antiplatelet therapy. A 600 mg loading dose was used because it is commonly used in clinical practice and provides the fastest and most-potent platelet inhibition with the use of clopidogrel [24]. Therefore, the present study adds important information about the lack of impact of a high-fat meal on clopidogrel-induced platelet aggregation measurement with some of the most commonly used assays.

Even though the present study provides novel information, inevitably there are still limitations. For one, the sample size in the present study was only calculated for LTA, thus the study may not have the power to detect differences in the other tests. Another limitation to the study is that it does not evaluate the effect of a high-fat meal on all platelet function assays such as multiplate analyzer and the thromboelastograph, of which neither were used in the present study. We also did not specifically collect data on body weight or body mass index for the subjects, which can be associated with poor responsiveness to clopidogrel. Finally, this study included young healthy volunteers instead of patients with known coronary artery disease or immediately after an acute coronary syndrome. While this is not expected to have had an impact on the results from the study, patients with active atherosclerotic disease are known to have higher levels of platelet aggregation.

## Conclusion

In the future, patients taking the P2Y_12_ antagonist clopidogrel could undergo an assessment of platelet function to determine appropriate dose or need for an alternative agent. It is imperative that the appropriate platelet assay is utilized under the appropriate conditions. In the present study, the intake of a high-fat meal did not significantly alter platelet function assessment of commonly used platelet function tests. Therefore, these data suggest that there is not a need to avoid a high-fat meal before platelet aggregation assessment. There was more intra-subject variability with the optically dependent compared with non-optically dependent platelet function tests, which will require further study.
